# Moderating effects of suicide resilience and meaning in life on the association between entrapment and suicidal ideation in Chinese patients with ovarian cancer: a cross-sectional study

**DOI:** 10.1186/s12888-023-05057-4

**Published:** 2023-08-11

**Authors:** Yinying Zhang, Xiaoping Ding, Jie Chen, Yilan Liu, Gang Wang, Deying Hu

**Affiliations:** 1 Department of Nursing, Union Hospital, Tongji Medical College, Huazhong University of Science and Technology, Wuhan, 430022 China; 2 School of Nursing, Tongji Medical College, Huazhong University of Science and Technology, Wuhan, 430030 China; 3 Wuhan Mental Health Center, Wuhan, 430010 China

**Keywords:** Ovarian cancer, Suicidal ideation, Suicide resilience, Meaning in life, Entrapment

## Abstract

**Background:**

Numerous studies have confirmed that patients with ovarian cancer have a relatively high risk of suicidality. Early identification of psychological factors related to suicidal ideation in patients with ovarian cancer may provide effective information for suicide prevention strategies. This study aimed to investigate whether and how suicide resilience and meaning in life moderate the relationship between entrapment and suicidal ideation in patients with ovarian cancer.

**Methods:**

This was a cross-sectional investigation was conducted in 505 Chinese patients with ovarian cancer. Patients completed a battery of self-reported questionnaires that included the General Information Questionnaire, and Chinese versions of the Entrapment Scale, Scale for Suicidal Ideation, Suicide Resilience Inventory-25, and Meaning in Life Scale. Descriptive statistics, Pearson’ s chi-square, Pearson’ s correlation, and hierarchical multiple linear regression analysis were performed.

**Results:**

In this study, the prevalence of suicidal ideation reported by patients with ovarian cancer was 32.07%. Patients’ suicidal ideation could be explained by the following three predictors: entrapment × suicide resilience × meaning in life (β = -0.169, *p* < 0.001), entrapment × suicide resilience (β = -0.148, *p* < 0.001), and entrapment × meaning in life (β = -0.107, *p* = 0.005).

**Conclusion:**

These findings suggest that ovarian cancer patients are prone to suicidal ideation when they feel a sense of entrapment. Enhancing patients’ suicide resilience and meaning in life may be two targeted interventions to reduce suicidal ideation in ovarian cancer patients. In particular, considering both the protective effects of suicide resilience and meaning in life may yield better suicide prevention outcomes than considering only one of these factors.

## Introduction

Ovarian cancer is the third-most common gynaecological cancer and most lethal gynaecological malignancy worldwide [1]. In 2022, it was estimated that 57,090 Chinese women would be diagnosed with ovarian cancer and 39,306 would die of the same [2]. Poor prognosis, low overall survival rate, surgical trauma, and long-term ongoing treatment can reduce the quality of life of ovarian cancer patients, leading to severe emotional distress, decreased self-esteem, and even suicidal ideation (SI) [3]. In the case of American women, it has been reported that those with gynaecologic malignancies exhibit a more than 12 times higher risk of suicide compared to those in the general population [4], and about a 1.3 times higher risk of suicide than those with non-gynaecologic malignancies [5]. Furthermore, numerous studies have confirmed that patients with ovarian cancer have the highest risk of suicidality across all gynaecological malignancies [3, 6–9].

Suicidality encompasses SI, suicide attempts, and completed suicide [10]. SI refers to any self-reported thoughts or planning of suicide and is likely to be an immediate precursor to a suicide attempt or completed suicide [11]. SI is a typical expression of suffering in cancer patients; it may indicate that the patient is suffering from severe depression, hopelessness, and loss of meaning in life [12]. When cancer patients express SI to healthcare professionals, it should first and foremost be interpreted as a cry for help, a sign of distress, or an attempt at seeking attention [13]. Therefore, early identification of factors related to SI in ovarian cancer patients may help relieve psychological suffering and provide effective information for suicide prevention strategies to prevent loss of life. A limited retrospective study investigated several general demographic and disease factors related to completed suicide among patients with ovarian cancer in the United States [3]. Although being aware of these factors is important, they may be limited in clinical practice applicability, especially for some non-modifiable factors (e.g., race and tumour histology). To date, little is known about the association between psychological factors and SI in patients with ovarian cancer.

The Integrated Motivational-Volitional (IMV) model of suicidal behaviour [14] proposes that the factors and processes resulting in the development of SI are different from those associated with suicidal behaviour (i.e. suicide attempts or completed suicide). Hence, this model clearly distinguishes the process of SI from suicidal behaviour across three stages: background factors or triggering events before the commencement of ideation formation (pre-motivational phase), formation of SI (motivational phase), and behavioural enaction (volitional phase). The IMV model’s motivational phase undergirds this study because it specifically explains the psychological processes leading to the development of SI and its related factors [14], which is helpful in providing insight into the processes and underlying mechanisms of SI among patients with ovarian cancer.

In the motivational phase, entrapment is the most proximal variable for SI [14]. Entrapment occurs when one desires to escape an adverse situation, but all escape routes are blocked [15]. Entrapment has been identified as a transdiagnostic psychological construct of SI among a range of high-risk populations, including psychiatric inpatients, sexual minorities, and combat veterans [16–18]. A large German study of 1529 cancer patients also reported that entrapment was linked to SI [19]. When individuals have experienced triggering events, negative thoughts or feelings concerning the event frequently come to mind, thereby inducing perceptions of entrapment [20]. Ovarian cancer is a major stressful life event, and patients may be likely to experience entrapping circumstances, resulting in the onset of SI [3].

The development of entrapment in SI is influenced by a set of motivational moderators (e.g., thwarted belongingness, burdensomeness, future thoughts, resilience, and meaning in life), which could increase or decrease the likelihood of moving from entrapment to SI [14]. This finding suggests that SI may not be an inevitable consequence of entrapment under the buffering effects of certain protective moderators [21]. Hence, future research must investigate the protective moderators that can weaken the relationship between entrapment and subsequent SI in patients with ovarian cancer. This study examined two important, but rarely explored, protective moderators: suicide resilience and meaning in life.

Resilience is a key factor in reducing SI and is increasingly regarded as a focus of suicide research and prevention [22]. To obtain a more accurate understanding of an individual’s resilience to suicide, researchers have proposed a relatively specific concept that expands upon general resilience and termed it suicide resilience [23]. Suicide resilience is defined as an individual’s perceived ability, resources, or competence to regulate suicide-related thoughts, feelings, and attitudes [24]. Improving suicide resilience among individuals at high risk of suicide may be a vital treatment target for mitigating SI risk [23]. Recently, there have been some suggestions that building suicide resilience in patients with cancer appears to be one of the most effective suicide prevention strategies [25]. The current literature examining the relationship between suicide resilience and SI in patients with ovarian cancer is limited, while some indirect evidence indicates that resilience may be a potential protective factor in reducing SI. A body of literature on patients with ovarian cancer has identified that low-level resilience is related to poorer quality of life, higher depressive symptoms, and anxiety [26–28], which are well-recognised risk factors for SI among patients with cancer [12, 29]. Although the IMV model proposes that resilience may be a protective moderator between entrapment and SI, empirical verification of the moderating role of suicide resilience is scarce. To the best of our knowledge, only one prior study has examined the moderating role of suicide resilience in the correlation between entrapment and SI among the general adolescent population [30]. Therefore, a replication study in ovarian cancer patients is needed, which will provide valuable information on effective prevention strategies for clinical samples at a high risk of SI.

Meaning in life may also be a protective moderator for SI. Meaning in life was first proposed by Frankl [31] and described as a therapeutic construct in which survivors search for meaning after a traumatic event. Meaning in life has become a current focus of cancer research, which has paid particular attention to its positive effects [32]. Meaning in life appears to be associated with a decrease in SI among cancer patients [33]. The diagnosis of ovarian cancer represents a significant threat, but acceptance of this difficult life challenge and pursuit of a meaningful life can improve patients’ positive emotions and tolerance of distress [34]. Based on the IMV model, a recent study of American college students provided promising evidence that meaning in life buffered the adverse effects of entrapment on SI [15]. Hence, our study sought to verify this finding among patients with ovarian cancer.

**Fig. 1:**
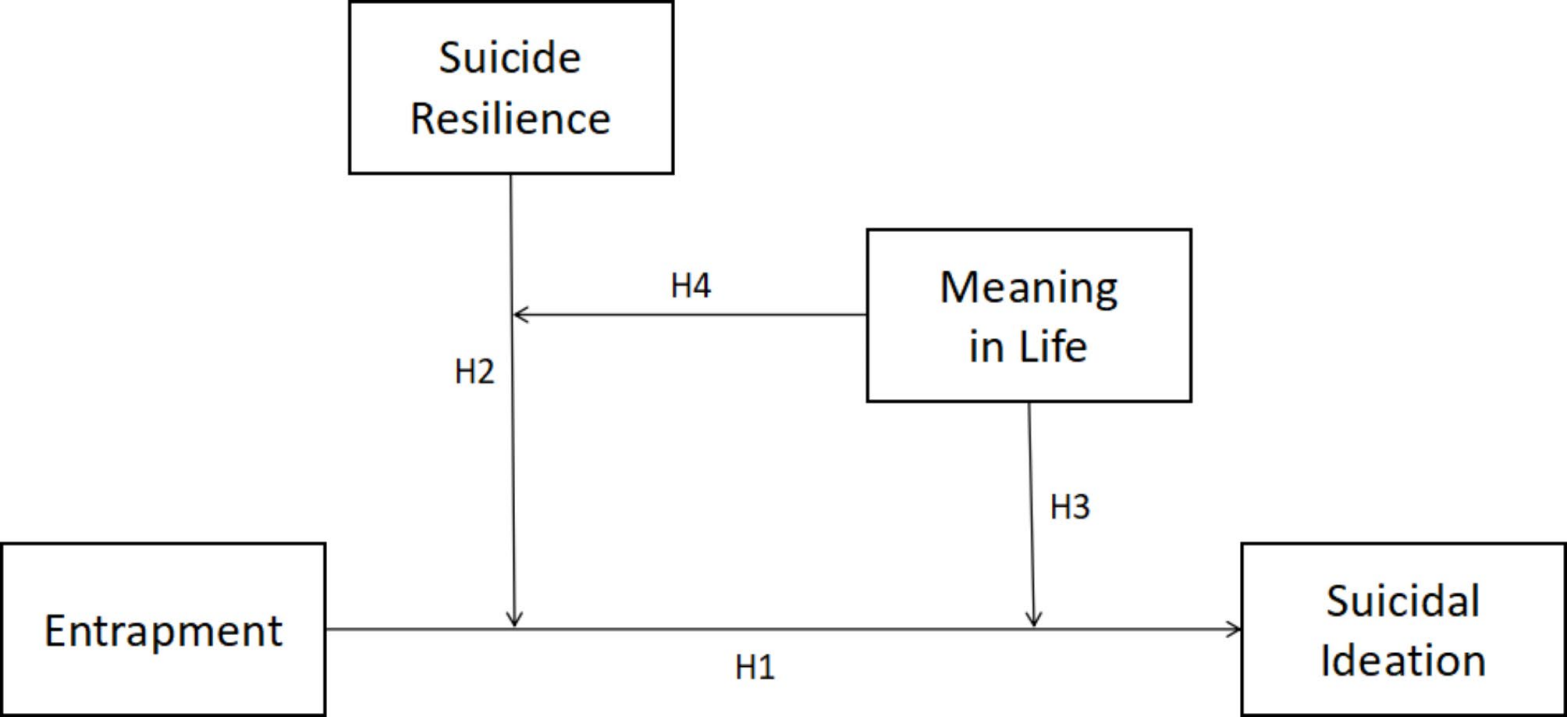
Theoretical model (H, hypothesis)

In summary, suicide resilience and meaning in life may be two crucial moderators that buffer the connection between entrapment and SI in patients with ovarian cancer. However, the interactions between suicide resilience, meaning in life, and entrapment in SI are not clear. According to previous research on the IMV model, a three-way interaction (moderator A × moderator B × entrapment) works better as a motivational moderator of SI than the two-way interactions (moderator A × entrapment or moderator B × entrapment) independently [35]. Therefore, the current study was conducted to investigate the separate moderating roles of suicide resilience and meaning in life (i.e. two-way interactions), and also attempts to examine whether and how suicide resilience and meaning in life would simultaneously moderate the relationship between entrapment and SI among Chinese patients with ovarian cancer (i.e. a three-way interaction). We propose the following hypotheses (Fig. 1):

H1: Entrapment is positively associated with SI.

H2: Suicide resilience moderates the relationship between entrapment and SI.

H3: Meaning in life moderates the relationship between entrapment and SI.

H4: Suicide resilience and meaning in life simultaneously moderate the relationship between entrapment and SI.

## Methods

### Design and participants

This cross-sectional study was conducted between March and October 2022. Participants were recruited from the Department of Gynaecological Oncology of four tertiary hospitals in Wuhan and Changsha, which are important institutions for treating patients with gynaecological cancer in central China. The median number of beds in the gynaecologic oncology departments across these four hospitals was 188 (interquartile range:135–196). Convenience sampling was used to recruit participants who met the following criteria: (i) histologically or cytologically confirmed ovarian cancer (tenth revision of the International Classification of Diseases, C56); (ii) age ≥ 18 years; (iii) history of chemotherapy, which is a high-risk factor for SI in patients with ovarian cancer [8]; and (iv) ability to communicate fluently in Chinese. Patients were excluded if they had severe cognitive dysfunction, critical illness, other comorbid cancers, or were unaware of their own cancer diagnosis.

The formula for sample size calculation for cross-sectional analysis was used: *N = Z*_*1−α/2*_^*2*^*× P(1-P) /d*^*2*^ [36], where *Z*_*1−α/2*_ equals 1.96 (at 5% type-I error), *d* represents the margin of error (0.15 was taken in this study), and *P* represents the prevalence of SI based on prior work (30.16%) [8]. Based on this formula, the required sample size was 405. Considering a 20% missing rate, the final sample size required for this study was 486.

### Data collection

Data collection was conducted by two researchers, both of whom were graduate students with experience in suicide prevention research in cancer populations, and supervised by a licensed consultant clinical psychologist. A pre-survey was administered to 30 ovarian cancer patients two weeks before formal data collection. The questionnaire response time was 15–30 min, which was moderate in length. The content of the questionnaire was clear and comprehensible to the participants. The researchers contacted the head nurses in advance to exclude patients who were unaware of their diagnosis because their families were reluctant to disclose the cancer diagnosis. Prior to the study, all participants were informed of its purpose and procedures and guaranteed that their personal information would be kept confidential and reported anonymously. Additionally, participants were told that upon completion of the questionnaire, they would receive 20 Chinese yuan (about 2.78 US dollars) as compensation for their time and participation in this study. After signing an informed consent form, participants were asked to complete a series of survey questionnaires. The researchers were in the same room as the participants throughout the completion of the questionnaire to offer explanations and assistance. During the data collection period, the research team verified the completeness of the information on all questionnaire daily and entered the data into Epidata version 3.1 (The EpiDate Association,Odense, Denmark). The survey was approved and continuously monitored by the Ethics Committee of XXX University and endorsed by each collaborating hospital (No. 2022-S015).

### Measures

The General Information Questionnaire was created based on a literature review of publications related to SI in patients with ovarian cancer [3, 8] and discussions among authors. It is a separate questionnaire that includes sociodemographic information and disease information. Self-reported information included age, marital status, employment status, and monthly per-capita family income. The disease information was obtained from the participant’s physician, including how long the diagnosis took, tumour histology, cancer stage, surgical history, presence of metastasis, and recurrence.

Entrapment was measured using the modified 16-item Chinese version of Entrapment Scale (C-ES) [37]. It contains two dimensions: external entrapment (10 items) and internal entrapment (6 items). Participants responded on a 5-point Likert scale (0 = not at all, 1 = a little bit, 2 = moderately, 3 = quite a bit, 4 = extremely). Higher scores indicate a stronger sense of entrapment. In this study, C-ES had a reasonable fit and level of acceptability (TLI was 0.94; CFI was 0.95; SRMR was 0.03; RMSEA was 0.06; and Cronbach’s α was 0.945).

SI was measured using the Scale for Suicide Ideation–Chinese Version (SSI-CV), initially developed by Beck et al. [38]. SSI-CV was a 19-item scale used to examine an individual’s SI experiences in their worst mood and/or the latest week. In this study, we investigated participants’ SI in the last week. SSI-CV was divided into two dimensions: assessment of suicidal ideation (the first five items) and severity of suicidal ideation (the last 14 items). The first five items included: (1) How much do you want to live? (2) How much do you want to die?3) Do your reasons for wanting to live outweigh your reasons for wanting to die?4) To what extent do you actively wish to attempt suicide? 5) To what extent do you wish to end your life externally, that is, have a “passive suicide wish”? (e.g., wish to stay asleep and never wake up, die unexpectedly, etc.). Each question was answered on a 3-point scale (0–2), with total scores ranging from 0 to 38. The higher the score, the worse the participant’s SI. Only participants who endorsed ‘weak’ or ‘moderate to strong’ levels of SI on items 4 or 5 were considered to have SI and completed the following 14 items, and their total score ranged from 1 to 38. Participants who endorsed ‘no’ levels of SI on items 4 and 5 were considered to have no suicidal ideation, and their total score ranged from 0 to 6. The scale’s good psychometric properties have been confirmed in the Chinese cancer population [39]. The Cronbach’s α for the SSI-CV was 0.958 in this study.

Suicide resilience was evaluated using the Chinese version of the Suicide Resilience Inventory-25 (C-SRI-25) [40]. The C-SRI-25 consists of three dimensions: emotional stability (e.g., “I can handle thoughts of killing myself when I feel lonely or isolated from other people”), internal protection (e.g., “I am proud of many good things about myself”), and external protective (e.g., “People close to me would find the time to listen if I were to talk seriously about killing myself”). A 6-point Likert scale is used to answer the 25 questions, yielding a total score ranging from 25 to 150 (1 = strongly disagree, 6 = strongly agree). Higher scores indicate greater protection against suicidal thoughts and behaviours. In this study, C-SRI-25 had a reasonable fit and acceptability (TLI was 0.92; CFI was 0.93; SRMR was 0.04; RMSEA was 0.06; and Cronbach’s α was 0.960).

Meaning in life was evaluated using the Chinese version of the Meaning in Life Scale (C-MiLS), which was developed specifically for patients with cancer [41]. This 25-item scale consists of five dimensions: acceptance and adaptation (six items), life perspective (six items), self-control (five items), relationships (five items), and purpose in life (three items). Each item of the C-MiLS is rated on a 5-point scale, from 1 = strongly disagree to 5 = strongly agree. Higher scores indicate greater perceptions of meaning in life. The scale has been validated in the Chinese cancer population and has shown good reliability and validity [41]. In this study, Cronbach’s α for the C-MiLS was 0.946.

### Data analysis

All data were analysed using the IBM SPSS software (version 25.0; SPSS Inc.). The analyses were completed in three stages. In the first stage, the presence or absence of SI was treated as a dichotomous variable for preliminary analysis of the differences that exist between the suicidal ideation and non-suicidal ideation groups. Suicidal ideation was coded as 0 = no suicidal ideation and 1 = suicidal ideation. In the second and third stages, the severity of suicidal ideation was treated as a continuous variable to analyse further the relationship between the severity of suicidal ideation and three psychological variables (entrapment, suicide resilience, and meaning in life).

In the first stage, descriptive statistics and Pearson’s chi-square test were used to describe and compare the differences between the suicidal ideation and non-suicidal ideation groups according to different sociodemographic and disease information. In the second stage, Pearson’s correlation analysis was used to measure the relationship between the four continuous variables (entrapment, suicide resilience, meaning in life, and SI). In the third stage, Hierarchical multiple linear regression analysis was performed to test how the independent variables (entrapment, suicide resilience, and meaning in life), two-way interactions (entrapment × suicide resilience, entrapment × meaning in life, and suicide resilience × meaning in life), and a three-way interaction (entrapment × suicide resilience × meaning in life) predicted the dependent variable (SI). The independent variables were mean-centred, and the two-way and three-way interactions were computed as the products of these mean-centred variables [42]. To examine the direction of significant interactions, a simple slope analysis was performed using a web page (http://www.jeremydawson.co.uk/slopes.htm) to interpret the effects of two-way and three-way interactions.

## Results

### Descriptive statistics and Pearson’s chi-square test

Of the 578 potential participants invited, 505 completed the questionnaires (87.3% participation rate), and 73 refused for the following reasons: physical discomfort (n = 23), too busy (n = 18), emotional reasons (n = 13), no interest (n = 9), caregiver refusal (n = 6), and other reasons (n = 4). In the current sample (n = 505), 162 participants (32.07%) reported SI. The average age of the participants was 55.19 (± 10.03) years (range 28–81). The majority of participants were aged 56 years or older (48.9%, n = 247), married (87.5%, n = 442), and unemployed (86.9%, n = 439). The monthly per-capita family income of 42.2% (n = 213) of participants was less than 1000 Chinese Yuan (≈ 148.30 US dollars), and 41.6% (n = 210) had been diagnosed with ovarian cancer less than six months ago. The tumour histology of most patients with ovarian cancer was serous (74.3%, n = 375), followed by mucinous (9.5%, n = 48), clear cell (5.9%, n = 30), endometrioid (4.0%, n = 20), or unknown (6.3%, n = 32). A total of 54.7% of the participants were diagnosed with stage III ovarian cancer (n = 276), 86.9% had surgical history (n = 439), 78.6% had metastasis present (n = 397), and 43.0% had recurrences (n = 217).

In addition, Pearson’s chi-square tests showed significant differences in ovarian cancer patients’ SI by marital status (*χ*^*2*^ = 15.828, *p* < 0.001), employment status (*χ*^*2*^ = 4.115, *p* = 0.043), monthly per-capita family income (*χ*^*2*^ = 29.766, *p* < 0.001), cancer stage (*χ*^*2*^ = 28.472, *p* < 0.001), metastasis present (*χ*^*2*^ = 13.232, *p* < 0.001), and recurrence (*χ*^*2*^ = 5.691, *p* = 0.017). Hence, these variables were included as covariates in the subsequent regression analyses. Bonferroni correction was used to examine the differences between groups and showed a higher prevalence of suicidal ideation in ovarian cancer patients who were single, unemployed, had monthly per capita family income < 1000, had cancer stage IV, and had metastasis present and recurrences. Table 1 presents further information.

**Table 1:** General information in participants with suicidal ideation and without suicidal ideation (n = 505)

Characteristics	Suicidal ideation			*χ* ^*2*^	*p*-value
	Total (n = 505)	With (n = 162)	Without (n = 343)		
Social-demographic information					
Age (years)				4.231	0.121
≤45	63 (12.5%)	18 (11.1%)	45 (13.1%)		
46 ~ 55	195 (38.6%)	54 (33.3%)	141 (41.1%)		
≥ 56	247 (48.9%)	90 (55.6%)	157 (45.8%)		
Marital status				15.828	**< 0.001**
Unmarried ^a^	63 (12.5%)	34 (21.0%)	29 (8.5%)		
Married	442 (87.5%)	128 (79.0%)	314 (91.5%)		
Employment status				4.115	**0.043**
Employed	66 (13.1%)	14 (8.6%)	52 (15.2%)		
Unemployed	439 (86.9%)	148 (91.4%)	291 (84.8%)		
Monthly per capita family income (yuan)^b^				29.766	**< 0.001**
< 1000	213 (42.2%)	95 (58.6%)	118 (34.4%)		
1000 ~ 3000	157 (31.1%)	43 (26.5%)	114 (33.2%)		
3001 ~ 5000	85 (16.8%)	14 (8.6%)	71 (20.7%)		
> 5000	50 (9.9%)	10 (6.2%)	40 (11.7%)		
Disease information					
Time to diagnosis (months)				4.017	0.260
< 6	210 (41.6%)	61 (37.7%)	149 (43.4%)		
6 ~ 12	80 (15.8%)	22 (13.6%)	58 (16.9%)		
13 ~ 36	124 (24.6%)	44 (27.2%)	80 (23.3%)		
> 36	91 (18.0%)	35 (21.6%)	56 (16.3%)		
Tumor histology				7.966	0.093
Serous	375 (74.3%)	133 (82.1%)	242 (70.6%)		
Mucinous	48 (9.5%)	10 (6.2%)	38 (11.1%)		
Clear cell	30 (5.9%)	6 (3.7%)	24 (7.0%)		
Endometrioid	20 (4.0%)	5 (3.1%)	15 (4.4%)		
Other/Unknown	32 (6.3%)	8 (4.9%)	24 (7.0%)		
Cancer stage ^c^				28.472	**< 0.001**
I	29 (5.7%)	3 (1.9%)	26 (7.6%)		
II	86 (17.0%)	14 (8.6%)	72 (21.0%)		
III	276 (54.7%)	91 (56.2%)	185 (53.9%)		
IV	114 (22.6%)	54 (33.3%)	60 (17.5%)		
Surgical history				0.267	0.605
Yes	439 (86.9%)	139 (85.8%)	300 (87.5%)		
No	66 (13.1%)	23 (14.2%)	43 (12.5%)		
Metastasis present				13.232	**< 0.001**
Yes	397 (78.6%)	143 (88.3%)	254 (74.1%)		
No	108 (21.4%)	19 (11.7%)	89 (25.9%)		
Recurrences					
Yes	217 (43.0%)	82 (50.6%)	135 (39.4%)	5.691	**0.017**
No	288 (57.0%)	80 (49.4%)	208 (60.6%)		

^a^ Unmarried includes those who are single, divorced or widowed

^b^1000 Chinese Yuan ≈ 148.30 US dollar; and the national per capita disposable annual income of Chinese residents in 2021 was 35,128 Chinese Yuan

^c^ cancer stage was diagnosed by clinical oncologists according to the 8th edition of the American Joint Committee on Cancer (AJCC) Staging System

### Pearson’s correlation analysis

As shown in Table 2, the Pearson’s correlation test revealed that SI had a significant positive correlation with entrapment (r = 0.501, *p* < 0.001), and significant negative relationships with suicide resilience (r = -0.490, *p* < 0.001) and meaning in life (r = -0.481, *p* < 0.001). Entrapment was negatively correlated with suicide resilience (r = -0.383, *p* < 0.001) and meaning in life (r = -0.477, *p* < 0.001). Suicide resilience was positively correlated with meaning in life (r = 0.382, *p* < 0.001). These four continuous variables showed skewness ranging from − 0.269 to 1.622 and kurtosis ranging from − 0.575 to 1.790 (Table 2). For samples larger than 300, absolute values of skewness and kurtosis less than two and seven, respectively, indicate a normal distribution [43].

**Table 2:** Pearson’s correlations analysis between entrapment, suicide resilience, meaning in life and suicidal ideation (n = 505)

Variables	1	2	3	4	Mean (SD)	Range	Skew	Kurt
1 Entrapment	1				17.73 ± 13.10	0–58	0.811	−0.149
2 Suicide resilience	−0.383	1			98.86 ± 19.74	35–145	−0.269	0.300
3 Meaning in life	−0.477	0.382	1		83.20 ± 18.53	36–118	−0.153	−0.575
4 Suicidal ideation	0.501	−0.490	−0.481	1	5.52 ± 7.66	0–38	1.622	1.790

All values statistically significant at p < 0.01 (two-tailed)

### Hierarchical multiple linear regression analysis

Hierarchical multiple linear regression was performed using a sequence of four steps (Table 3). In Step 1, we entered six covariates into the regression, including marital status, employment status, monthly per-capita family income, cancer stage, metastasis, and recurrence. The results suggested that the covariates explained 13.6% of the variance in SI. In Step 2, entrapment, suicide resilience, and meaning in life were significant predictors of SI, accounting for 30.1% of the additional variance. In Step 3, both the interaction between entrapment and suicide resilience and that between entrapment and meaning in life were significant. However, the interaction between suicide resilience and meaning in life was not significant. The two-way interactions predicted an additional 3.2% of the variance in SI. In Step 4, the three-way interaction between entrapment, suicide resilience, and meaning in life was significant, accounting for 1.6% of the additional variance. Patients’ SI could be explained by the following three predictors: entrapment × suicide resilience × meaning in life (β = -0.169, *p* < 0.001), entrapment × suicide resilience (β = -0.148, *p* < 0.001), and entrapment × meaning in life (β = -0.107, *p* = 0.005). Finally, the regression explained 47.0% (adjusted R^2^) of the total variance in SI among patients with ovarian cancer. Residual plots were generated after the regression models and showed an approximately normal distribution. Regression diagnostics suggested no serious multicollinearity issues (variance inflation factor < 1.819) for any variable.

**Table 3:** Hierarchical multiple linear regression analysis testing the moderating effects of suicide resilience and meaning in life on the relation between entrapment and suicidal ideation (n = 505)

Variables	Model 1		Model 2		Model 3		Model 4	
	*B*	t	*B*	t	*B*	t	*B*	t
Step1: Covariates								
Marital status	−0.152***	−3.563	−0.119**	−3.435	−0.102**	−3.024	−0.090**	−2.674
Employment status	−0.028	−0.573	0.003	0.082	0.001	0.028	0.000	0.003
Monthly per capita family income	−0.195***	−3.972	−0.054	−1.320	−0.055	−1.371	−0.058	−1.479
Cancer stage	0.203***	3.830	0.112*	2.576	0.117**	2.754	0.105*	2.493
Metastasis present	−0.009	−0.167	−0.001	−0.032	0.010	0.240	0.008	0.188
Recurrences	−0.027	−0.616	−0.046	−1.288	−0.030	−0.876	−0.020	−0.580
Step2: Independent variables								
Entrapment			0.263***	6.587	0.216***	5.304	0.240***	5.910
Suicide resilience			−0.281***	−7.435	−0.272***	−7.340	−0.334***	−8.367
Meaning in life			−0.194***	−4.752	−0.202***	−5.050	−0.226***	−5.661
Step 3: Two-way interactions								
Entrapment × Suicide resilience					−0.110**	−2.684	−0.148***	−3.572
Entrapment × Meaning in life					−0.075*	−2.005	−0.107**	−2.841
Suicide resilience × Meaning in life					0.050	1.274	0.043	1.094
Step 4: Three-way interaction								
Entrapment × Suicide resilience × Meaning in life							−0.169***	−3.857
R^2^	0.136		0.436		0.468		0.483	
Adjusted R^2^	0.125		0.426		0.455		0.470	
ΔR^2^	0.136		0.301		0.032		0.016	
F	13.014		42.531		36.031		35.342	

B, standardized regression coefficient; SE, standard error; R^2^, R-squared; ΔR^2^, delta R-squared

* P < 0.05;** P < 0.01;*** P < 0.001

**Fig. 2:**
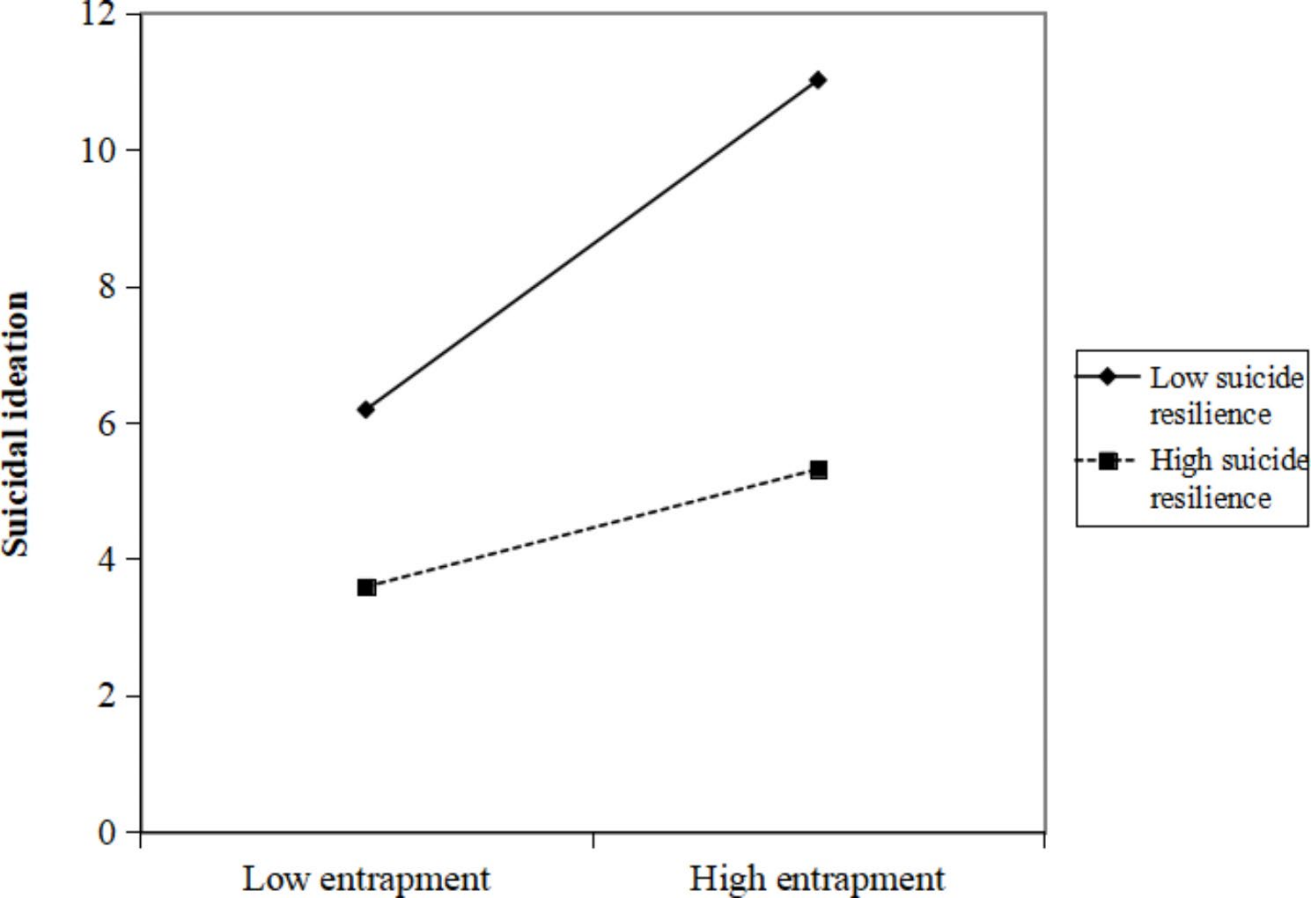
Suicide resilience moderates the relationship between entrapment and suicidal ideation

**Fig. 3:**
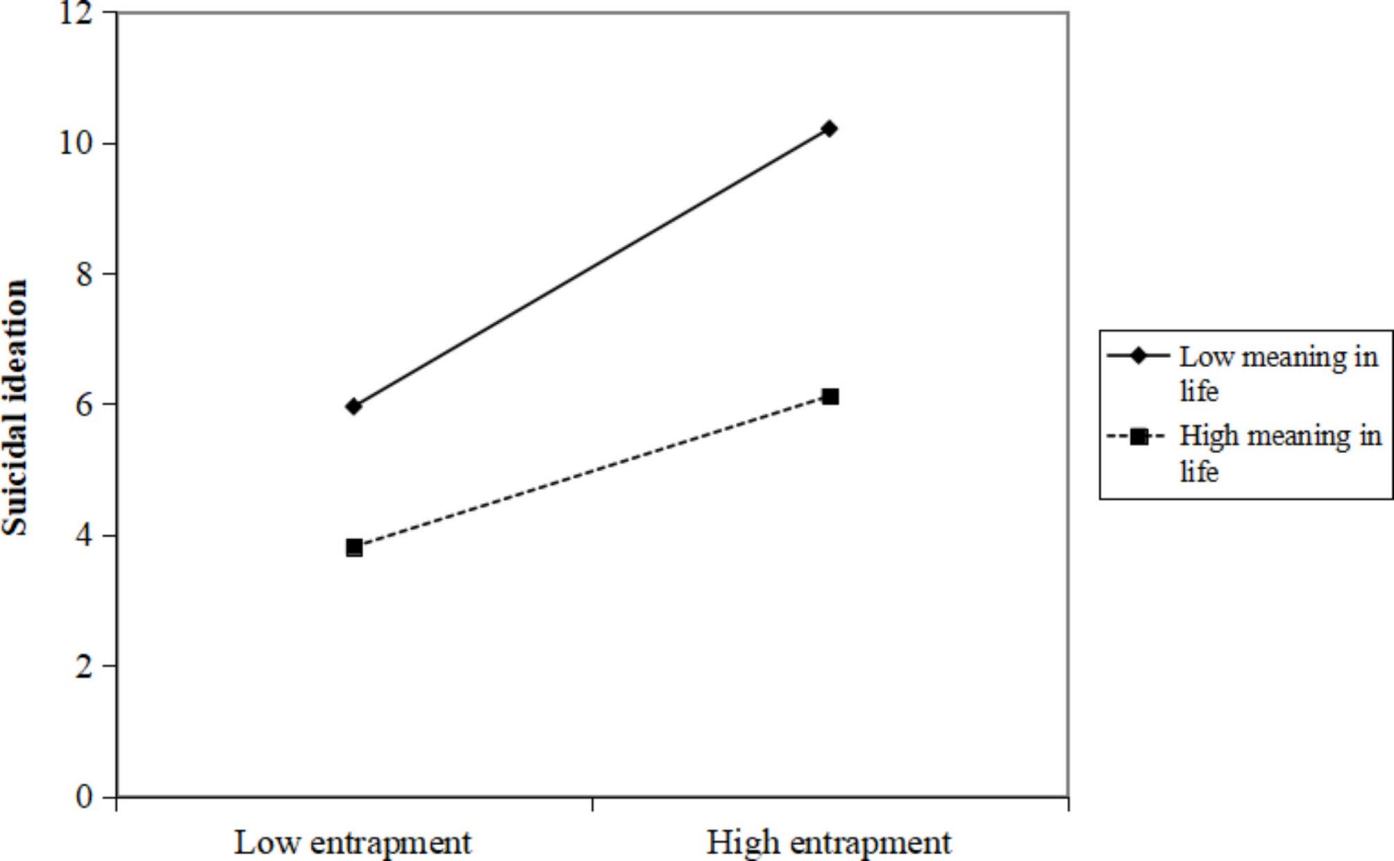
Meaning in life moderates the relationship between entrapment and suicidal ideation

**Fig. 4:**
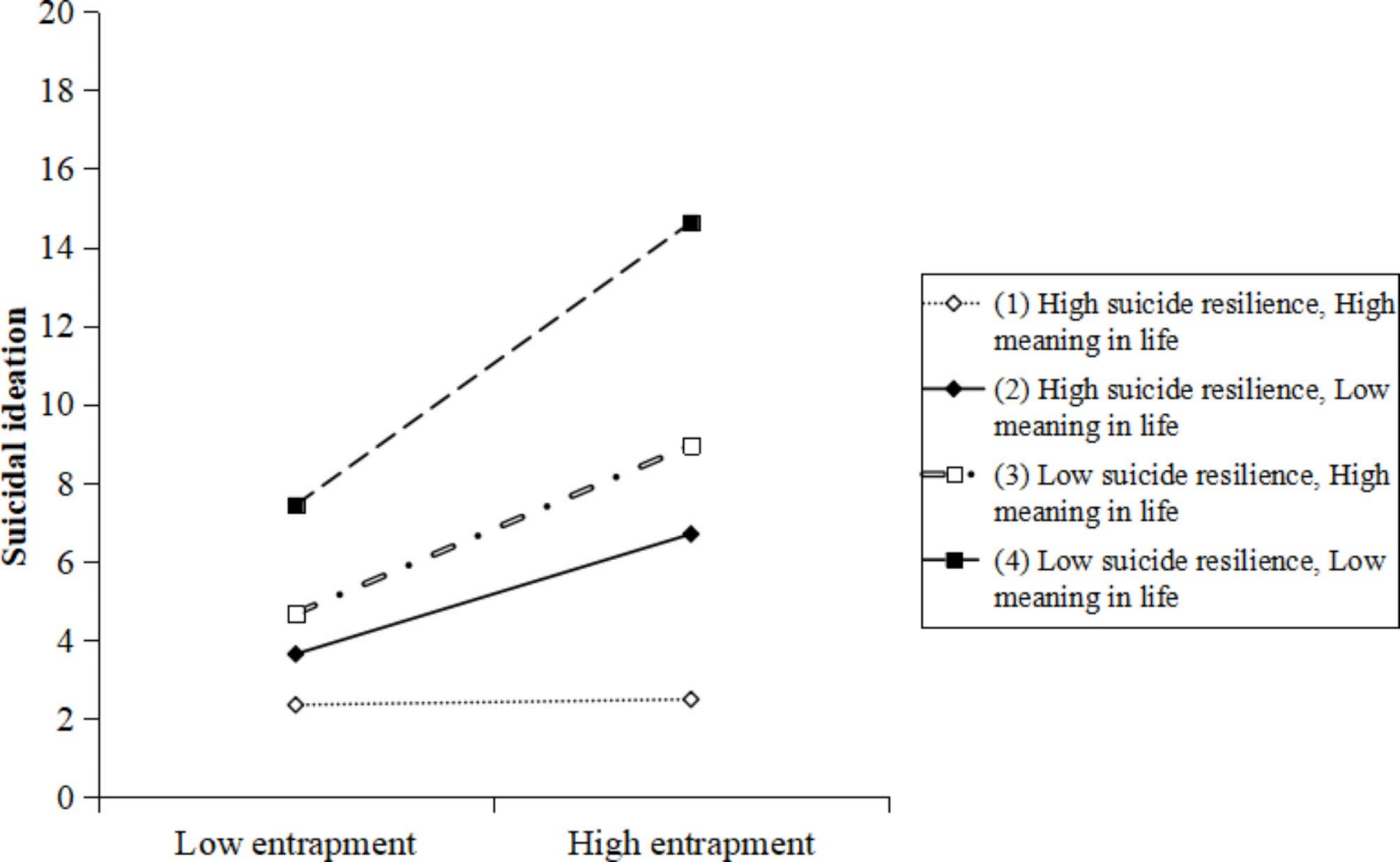
Suicide resilience and meaning in life moderates the relationship between entrapment and suicidal ideation

According to the simple slope method for checking interactions in multiple regression [42], one standard deviation above the mean was considered as high categories of suicide resilience, meaning in life, and entrapment; and one standard deviation below the mean was considered a low category of suicide resilience, meaning in life, and entrapment. Figures 2, 3 and 4 depict the simple slopes at high (i.e. +1SD) and low (i.e. −1SD) levels of entrapment. As shown in Fig. 2, when ovarian cancer patients had a low level of suicide resilience, there was a significant positive relationship between entrapment and SI (t = 5.361, *p* < 0.001). However, this positive relationship between entrapment and SI was not significant for patients with high suicide resilience (t = 1.698, *p* = 0.090). As demonstrated in Fig. 3, there were significant positive relationships between entrapment and SI when ovarian cancer patients had a low level of meaning in life (t = 4.611, *p* < 0.001) or a high level of meaning in life (t = 2.250, *p* = 0.025). As shown in Fig. 4, when ovarian cancer patients exhibited a low level of suicide resilience and low level of meaning in life, there was a significant positive relationship between entrapment and SI (t = 7.772, *p* < 0.001). This positive relationship between entrapment and SI was still significant, but much weaker for patients with low suicide resilience and high meaning in life (t = 3.419, *p* = 0.001) and high suicide resilience with low meaning in life (2.427, *p* = 0.016). However, entrapment did not significantly predict SI among ovarian cancer patients when their suicide resilience and meaning in life were both high (t = 0.112, *p* = 0.911).

## Discussion

In this study, the prevalence of SI reported by Chinese women with ovarian cancer was 32.07%, which was much higher than that reported among the Chinese female population (4.9%) [44] and other Chinese female cancer survivors (17.6%) [45]. Another study found a similar prevalence of SI (30.16%) in Chinese patients with ovarian cancer [8]. This finding confirms the large number of previous studies indicating that patients with ovarian cancer are at a high risk of suicidality [3, 6–9]. Although some researchers have highlighted the importance of regular screening for SI in the cancer population, in actual clinical practice, healthcare professionals pay far less attention to SI in cancer patients, especially ovarian cancer patients [12]. This may be due to the relative lack of psychiatry professional in Chinese oncology hospitals [46]. Therefore, enhanced communication and collaboration between psychiatrists and oncologists is necessary to maximize patient safety.

The present study found a positive association between entrapment and SI in ovarian cancer patients, consistent with previous validation in other populations, such as veterans [16–18]. Entrapment is one of the main psychological symptoms in patients with cancer, indicating a sense of social isolation and deep distress [19]. Women with ovarian cancer may experience severe feelings of social isolation. For example, some patients report that they experience impaired intimacy with their spouses following the ovarian cancer diagnosis because of sexual dysfunction [47]. Some ovarian cancer patients state that they have lost close friends because they recognise that their friends feel uncomfortable talking about their condition [48]. Additionally, ovarian cancer patients may also experience deep distress, probably because multiple recurrences are common among ovarian cancer patients, resulting in a huge financial burden, exhaustion due to prolonged hospitalisation, and severe mental distress [49]. SI occurs when suicide is seen as the only way for patients with ovarian cancer to escape from an aversive entrapped state [14]. When ovarian cancer patients report SI to healthcare providers, they may express a feeling of being trapped and a desire for help [13]. Hence, healthcare providers should recognise the potential mechanism linking entrapment and SI in patients with ovarian cancer, so they can help patients escape these circumstances.

This study also verified that suicide resilience played a moderating role between entrapment and SI in patients with ovarian cancer. This conclusion supports previous studies’ findings that suicide resilience moderates the relationship between entrapment and SI in adolescents [30]. As a positive mental health resource, promoting suicide resilience is an important strategy to reduce SI because resilient ovarian cancer patients generally have fewer complaints and act positively and dynamically to combat adversity [23, 27]. The simple slope results indicated that entrapment was positively associated with SI when patients with ovarian cancer had a low level of suicide resilience. Interestingly, this relationship was not significant for ovarian cancer patients with a high level of suicide resilience. These findings are consistent with a previous report by Li et al. that reported entrapment was positively associated with SI only at a low level of suicide resilience [30]. Suicide resilience means that a person possesses both internal and external resources to buffer a series of negative life events, thereby moderating SI [24]. Ovarian cancer patients with high levels of suicide resilience can recover from negative life events, even if they feel trapped. Therefore, they are less likely to see suicide as the only way out of entrapment.

Consistent with previous studies, this study found that meaning in life moderated the relationship between entrapment and SI [15]. Patients with ovarian cancer generally have a lower quality of life throughout the disease cycle; helping them find meaning in their entrapped life may be an important means of reducing their risk of suicide [50]. A large randomised controlled study conducted by Breitbart et al. [33] provided evidence that patients with advanced cancer receiving meaning-centred group psychotherapy showed significant reductions in desire for hastened death. The simple slope analysis showed a significant positive correlation between entrapment and SI when ovarian cancer patients had either low or high levels of meaning in life. The diagnosis of ovarian cancer is a negative life event that may change a patient’s previous perceptions of their life. When patients are entrapped, their sense of helplessness and incompetence worsens, leading to a complete loss of meaning in life and, in severe cases, to suicidal thoughts [51]. In contrast, patients with high meaning in life gradually realise the value of life and importance of health as a result of their long battle with the disease; hence, they are able to escape from entrapment and live actively with their illness [52]. Notably, individuals’ perspectives on meaning in life may vary with cultural background [40]. Influenced by Confucian culture, Chinese women with cancer believe that they can experience meaning in life if they successfully live with cancer and continue to contribute to their families, especially by taking care of their husbands and children [53]. Healthcare professionals should understand the impact that cultural differences may have on the meaning in life for ovarian cancer patients, guiding them to discover the joy of life, encouraging them to cherish life, and ultimately achieve the goal of suicide prevention.

Additionally, this study demonstrated a three-way interaction effect in which suicide resilience and meaning in life could synergistically moderate the relationship between entrapment and SI. Interestingly, we found that the three-way interaction (i.e. entrapment × suicide resilience × meaning in life) works better as a motivational moderator of SI than the two-way interactions independently (i.e. entrapment × suicide resilience or entrapment × meaning in life). This result suggests that considering both the protective effects of suicide resilience and meaning in life among patients with ovarian cancer may yield better suicide prevention outcomes. Furthermore, the slope analysis explained how suicide resilience and meaning in life interacted to affect the relationship between entrapment and SI. We found that higher levels of entrapment significantly predicted increases in SI when suicide resilience and meaning in life were either both low, or one was low. Specifically, our results revealed the strongest relationship between entrapment and SI when both suicide resilience and meaning in life were low (t = 7.772, *p* < 0.001). This was followed by patients with low suicide resilience and high meaning in life (t = 3.419, *p* = 0.001) and patients with high suicide resilience and low meaning in life (t = 2.427, *p* = 0.016). This suggests that healthcare professionals should give high priority to the possibility of SI in patients with ovarian cancer when they present with low suicide resilience, regardless of whether their meaning in life is high or low. As a vulnerability factor, low suicide resilience is a strong predictor for SI [24]. Fostering suicide resilience in patients with ovarian cancer may be a valuable intervention to reduce the risk of suicide because suicide resilience is not static; instead, it is dynamic [23]. A growing body of literature points to an increased understanding of meaning in life as an important method to improve individuals’ suicide resilience [23, 54]. This speaks to the complicated nature of suicide resilience and interactions in meaning in life. However, entrapment was not associated with SI when patients’ suicide resilience and meaning in life were both high. A recent study found that general population with high levels of both resilience and meaning in life had good mental health and a positive perception of the past [55]. Ovarian cancer patients with high levels of both suicide resilience and meaning in life are highly likely to have positive thoughts during their experience with cancer and define the cancer event as less threatening [56]. Consequently, they may be less likely to experience entrapment and SI.

Our study likewise identified many general demographic (single, unemployed, household per capita monthly income < 1000) and disease factors (stage IV cancer, presence of metastases and recurrence) associated with suicidal ideation in ovarian cancer patients. Single ovarian cancer patients lack better family emotional and financial support and suffer more psychological stress than married ones [57]. Ovarian cancer patients with poor economic status have significantly lower quality of life in several domains including physical, social and emotional functioning [58]. These several socioeconomic-related general demographic factors (singleness, unemployment, low household income) may place ovarian cancer patients in a malignant psychological environment, which increases their risk of suicidal ideation. In addition, the characteristics of ovarian cancer disease itself may also be responsible for increasing their risk of suicidal ideation. Due to the insidious early symptoms and the lack of practical screening tools, ovarian cancer is most advanced at initial diagnosis [59]. Ovarian cancer is highly susceptible to recurrence and metastasis, and the 5-year survival rate of ovarian cancer patients is roughly 50% [60]. When an ovarian cancer patient is in stage IV cancer, or have metastasis or recurrence, their quality of life may be lower, their emotional problems more severe, and their risk of subsequent suicidal ideation may increase [3]. These meaningful findings may help medical professionals to identify at-risk groups for suicidal ideation in ovarian cancer patients as early as possible in their clinical work. The professionals could provide appropriate psychosocial support to these high-risk groups to reduce their emotional distress and thus reduce their risk of suicidal ideation.

### Study strengths and limitations

To the best of our knowledge, this is the first study to specifically investigate the relationship between SI and its related psychological factors in patients with ovarian cancer. Screening and assessing the psychological factors of SI in patients with ovarian cancer may provide valuable information to develop suicide prevention strategies. These findings suggest that ovarian cancer patients are prone to SI when they feel a sense of entrapment. Enhancing patients’ suicide resilience and meaning in life may be two targeted interventions to reduce SI in ovarian cancer patients. For patients with low suicide resilience, healthcare professionals can teach them techniques to maintain emotional stability if they experience suicidal thoughts and should encourage patients to seek help from family members and psychiatrist if SI occurs. It is also important to enhance the meaning of life for patients with ovarian cancer. Healthcare professionals must help patients form the correct perspective on illness and death and explore the meaning of life from a positive perspective.

Our study also has some limitations. First, this was a cross-sectional design; thus, causality between the variables and the time sequence of their occurrence cannot be established. However, our study provides a basis upon which future intervention studies may expand, because our proposed relationship between variables was based on theoretical evidence and supported by empirical data. Second, our study relied on self-reported measurements. Therefore, ‘self-report bias’ may have occurred if the participants presented themselves in a more favourable light because of the shame they felt about having suicidal thoughts. Finally, patients with ovarian cancer who refused to participate in this study may have experienced severe physical or emotional distress, which could have led to selection bias. We were unable to compare the differences in sociodemographic and disease information between the participants who declined and those who completed the survey because we were unable to collect this information.

## Conclusion

This study confirmed that both suicide resilience and meaning in life can buffer the adverse effects of entrapment on SI in Chinese patients with ovarian cancer. More importantly, our findings suggest that considering both the protective effects of suicide resilience and meaning in life may yield better suicide prevention outcomes than considering only one of these factors. In particular, the strongest relationship between entrapment and SI was when both suicide resilience and meaning in life were low. Our findings could be helpful in identifying related factors in ovarian cancer patients with SI, which may play an important role in future suicide prevention programmes.

## Data Availability

Due to restrictions e.g. privacy or ethical, data are available from the corresponding author upon reasonable request.

## Abbreviations

SI: Suicidal ideation
IMV: Integrated Motivational-Volitional
C-ES: Chinese version of Entrapment Scale
SSI-CV: The Scale for Suicide Ideation–Chinese Version
C-SRI-25: The Chinese version of the Suicide Resilience Inventory-25
C-MiLS: The Chinese version of the Meaning in Life Scale
